# Comprehensive characterization of *TGFB1* across hematological malignancies

**DOI:** 10.1038/s41598-023-46552-8

**Published:** 2023-11-04

**Authors:** Cui-zhu Wang, Zi-qi Zhang, Yan Zhang, Liang-feng Zheng, Yang Liu, Ai-ting Yan, Yuan-cui Zhang, Qing-hua Chang, Suo Sha, Zi-jun Xu

**Affiliations:** 1 Department of Hematology and Oncology, Affiliated Haian Hospital of Nantong University, Nantong, China; 2 School of Medicine, Jiangsu University, Zhenjiang, China; 3 Laboratory Center, Affiliated Haian Hospital of Nantong University, Nantong, China; 4 Clinical Nutrition Department, Haian Hospital of Traditional Chinese Medicine, Nantong, China; 5 Department of Respiratory Medicine, The Affiliated Zhenjiang Third Hospital of Jiangsu University, 1 Dingmao Bridge, Youth Square, Zhenjiang, 212002 China; 6 Surgery of Traditional Chinese Medicine, Haian Hospital of Traditional Chinese Medicine, Nantong, 226600 China; 7 Laboratory Center, Affiliated People’s Hospital of Jiangsu University, 8 Dianli Rd., Zhenjiang, 212002 China

**Keywords:** Cancer, Computational biology and bioinformatics, Immunology

## Abstract

*TGFB1*, which encodes TGF-β1, a potent cytokine regulating varies cellular processes including immune responses. TGF-β1 plays context-dependent roles in cancers and is increasingly recognized as a therapeutic target to enhance immunotherapy responses. We comprehensively evaluated expression of *TGFB1* and its clinical and biological effects across hematological malignancies. *TGFB1* expression was first explored using data from the GTEx, CCLE, and TCGA databases. The expression and clinical significances of *TGFB1* in hematological malignancies were analyzed using Hemap and our In Silico curated datasets. We also analyzed the relationship between *TGFB1* with immune scores and immune cell infiltrations in Hemap. We further assessed the value of *TGFB1* in predicting immunotherapy response using TIDE and real-world immunotherapy datasets. *TGFB1* showed a hematologic-tissue-specific expression pattern both across normal tissues and cancer types. *TGFB1* expression were broadly dysregulated in blood cancers and generally associated with adverse prognosis. *TGFB1* expression were associated with distinct TME properties among different blood cancer types. In addition, *TGFB1* expression was found to be a useful marker in predicting immunotherapy responses. Our results suggest that *TGFB1* is broadly dysregulated in hematological malignancies. *TGFB1* might regulate the immune microenvironment in a cancer-type-specific manner, which could be applied in the development of new targeted drugs for immunotherapy.

## Introduction

Transforming growth factor β1 (TGF-β1) is a potent cytokine capable of regulating multiple cellular processes including cell proliferation, differentiation, wound healing, and immune response. TGF-β1 is identified in various immune cells and its activity varies in different cell types or cells at different developmental stages^1^. TGF-β1 functions by binding to the TGFβ1 receptors, which subsequently activate the canonical SMAD pathway or non-canonical signaling cascades, such as the mitogen-activated protein kinase (MAPK) pathway, phosphatidylinositol-3 kinase and AKT (PI3K-AKT), and Rho family of GTPases (Rho GTPase)^2^. What is special about TGF-β1 is its context-dependent nature that is particularly true for tumors. Generally, in healthy epithelial tissues and during the early stages of tumorigenesis, TGF-β1 negatively regulates the proliferation and growth on premalignant epithelial cells and thus suppresses tumor progression. Conversely, in late-stage cancers, tumor cells could rewire the TGFβ1 pathway to avoid apoptosis and suppress immune responses, which promotes tumor progression^3^. Also, the opposing effects of TGF-β1 depend on cancer types and even cancer subtypes^4^.

It is worth noting that TGF-β1 may mediate pro- and anti-tumor effects through both cell-intrinsic and -extrinsic factors; it might orchestrate the tumor microenvironment (TME) by promoting tumor-promoting components like cancer-associated fibroblasts (CAF)-like cells^5^ and suppressing cytotoxic cells like CD8 + T cells^6,7^. Indeed, TGF-β1 has already been experimentally exploited as a therapeutic target to enhance responses to immunotherapy^8,9^. In hematological malignancies, for example, TGFβ signaling has been studied as a therapeutic target to treat the ineffective erythropoiesis in lower risk myelodysplastic syndromes (LR-MDS) patients^10^. While immunotherapy-especially immune checkpoint blockade-seems to have limited effect on blood cancers, TGFβ1 was shown to be deeply involved in the pathogenesis of these cancer types^11,12^. However, there have been no comprehensive studies on TGFβ in a pan-blood-cancer level to date. In this study, we comprehensively evaluated the expression of *TGFB1* (coding gene of TGFβ) and its clinical and biological effects across multiple blood cancer types. We also explored the association between *TGFB1* and the immune cell infiltration, immune genes, and immunotherapy responses. Our results provide novel insights into the functional role of *TGFB1* in hematological malignancies and the potential as cancer immunotherapy targets.

## Methods

### Data collection

The mRNA expression data of normal tissues and cancer cell lines were obtained from Genotype-Tissue Expression (GTEx) project (www.gtexportal.org/) and Cancer Cell Line Encyclopedia (CCLE) (https://www.broadinstitute.org/ccle), respectively. The curated Hemap dataset comprising 16 major blood cancer types and normal blood cell types were defined by Dufva O et al. (Synapse ID: syn21991014; DOI: https://doi.org/10.7303/syn21991014)^13^. The combined pan-cancer data of TCGA, TARGET, and GTEx were downloaded from UCSC Xena Browser (https://xenabrowser.net). Additionally, we curated the Pan-Hem-Diff cohort which composed of 40 datasets with matched tumor and normal samples encompassing 22 blood cancer types (n = 9101). We also comprehensively searched datasets for acute myeloid leukemia (AML), diffuse large B-cell lymphoma (DLBCL), and multiple myeloma (MM) with survival information from the following sources: GEO database (https://www.ncbi.nlm.nih.gov/geo/), GDC data portal (https://portal.gdc.cancer.gov/), cBioPortal for Cancer Genomics (http://www.cbioportal.org/), and PREdiction of Clinical Outcomes from Genomic Profiles (PRECOG, https://precog.stanford.edu/). Copy number variation (CNV) data and mutation data of blood cancers were gathered from the cBioPortal for Cancer Genomics (http://www.cbioportal.org/). We used the methylation data downloaded from GEO database (https://www.ncbi.nlm.nih.gov/geo/) to analyze the methylation status of *TGFB1* in blood cancers. All the datasets utilized in this study were summarized with their accession numbers and usages in Table S1.

### Single-cell sequencing analysis

Single-cell RNA-seq (scRNA-seq) data of blood cancers were curated by Tumor Immune Single-cell Hub 2 (TISCH2) (http://tisch.comp-genomics.org/home/)^14^. The expression data of *TGFB1* was downloaded and reproduced with ggplot2. The data matrix contained *TGFB1* expression data from seven blood cancer types with 25 scRNA-seq datasets and 19 cell types.

### Tumor immune microenvironment analysis

29 functional gene expression signatures regarding various aspects of TME were retrieved from previous study^15^ and the signature scores were calculated using single-sample gene set enrichment analysis (ssGSEA). The immune scores and stromal scores for each sample were calculated using both the ESTIMATE and xCELL algorisms. CIBERSORT and MCP-counter algorithms was applied to estimate the relative fractions of infiltrating immune cell types in each blood cancers. The cytolytic score was originally calculated using the Hemap dataset by Dufva O et al.^13^.

### Immunotherapy response analysis

The potential responses to immunotherapy in datasets covering five blood cancer types were predicted by the TIDE algorithm. We also collected real-world transcriptomics data of patients treated with immunotherapies. The transcriptomic data and response information of these studies were obtained from GEO, TIDE platform, or from the original publications. The accession number or source of each study was summarized in Table S1.

### Survival analysis

Kaplan–Meier analysis was performed to evaluate the overall survival (OS), progression-free survival (PFS), and event-free survival (EFS) of patients. The Survminer package were used to determine of best cut-off of *TGFB1* expression and produce Kaplan–Meier survival plots. Univariate Cox regression analyses were conducted to assess the significance of *TGFB1* in predicting OS in three blood cancers. Then we performed meta-analyses to combine *p* values and hazard ratios (HRs) using the survcomp package. The LSC17 and LI24 prognostic models were constructed as previously described^16,17^.

### Gene set enrichment analyses

To ensure that more meaningful biological interpretations could be derived from multiple blood cancer types, we used a more stringent criteria to dichotomize *TGFB1* expression across cancer types. We calculated differentially expressed genes (DEGs) between the top 30% *TGFB1* expression subgroup and bottom 30% *TGFB1* expression subgroup in each blood cancer type in Hemap. DEGs between the high- and low- *TGFB1* subgroups were selected for gene set enrichment analysis (GSEA). GSEA was performed using the R package clusterProfiler. The Hallmarks gene set from MySigDB (http://www.broad.mit.edu/gsea/msigdb) were selected for GSEA. We also used the GSVA package to compute signature scores in the GSE116256 scRNA-seq dataset. Differential analyses of the gene expression or signature score data were performed using the limma package.

### Statistical analyses

Differences between groups were analyzed using Wilcoxon rank sum tests for continuous variables. For differential gene expression results derived from Pan-Hem-Diff, the *p*-values and log-fold changes (FCs) for datasets belonging to the same cancer type were further combined using the MetaVolcanoR package. Chi-square tests or Fisher's exact tests were used to compare differences between categorical variables. Spearman correlation analysis was used to determine the correlation between two continuous variables. All statistical analyses were performed using the R software, with most plots produced using the ggplot2 package. *p* < 0.05 (two-tailed) was considered statistically significant.

## Results

### Landscape of expression of *TGFB1* in normal tissues and across cancer types

**Figure 1:**
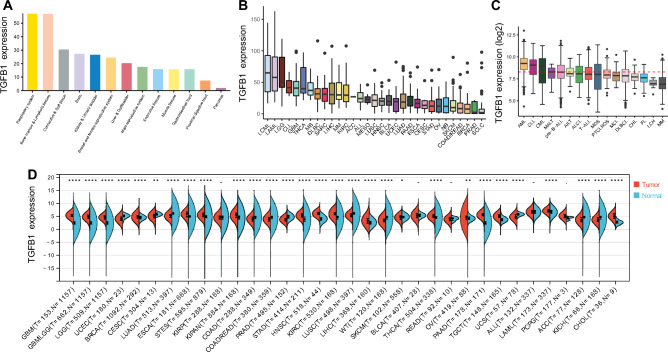
Landscape of expression of *TGFB1* in normal tissues and across cancer types. (**A**) Bar plot showing mRNA expression levels of *TGFB1* in normal tissues from the Genotype-Tissue Expression (GTEx) database. (**B**) Boxplot showing mRNA expression levels of *TGFB1* in various tumor cell lines from the Cancer Cell Line Encyclopedia (CCLE) database. (**C**) Boxplot showing mRNA expression levels of *TGFB1* across main blood cancer types in Hemap. The dotted red line indicates median value of *TGFB1* expression. (**D**) Boxplot showing mRNA expression differences of *TGFB1* between tumor and normal tissue samples, combining data from TCGA, TARGET, and GTEx databases. Blue, normal control samples; red, tumor samples. *p < 0.05; **p < 0.01; ***p < 0.001; -Not significant.

We first determined the expression level of *TGFB1* in normal tissues based on the GTEx database (http://www.GTExportal.org/home/). The top two *TGFB1*-enriched tissues were respiratory system and bone marrow & lymphoid tissues (Fig. 1A). Analyzing *TGFB1* expression in normal cell types from Hemap revealed preferential *TGFB1* expression in myeloid cell fractions, such as monocytes, neutrophils, and macrophages (Figure S1A). Next, using CCLE, we showed that *TGFB1* were highly expressed in malignant hematological cell lines from chronic myeloid leukemia (CML), AML, chronic lymphocytic leukemia (CLL), and DLBCL (Fig. 1B). These results prompted us to investigate *TGFB1* expression across hematological malignancies. In Hemap, higher *TGFB1* expression in AML, CLL, and CML was again observed (Fig. 1C). Overall, these findings indicated a cellular-, tissue-, and disease- specific *TGFB1* expression. Combining GTEx dataset with TCGA and TARGET pan-cancer datasets, we then systematically compared *TGFB1* expression between tumor and adjacent normal tissue across 34 cancer types. Surprisingly, *TGFB1* were significantly dysregulated in almost all cancer types: it was significantly up-regulated in 17 cancer types such as AML, breast invasive carcinoma (BRCA), and cholangiocarcinoma (CHOL), whereas it was significantly down-regulated in 12 cancer types (Fig. 1D).

### Landscape of expression and (epi)genetic alterations of *TGFB1* across blood cancer types

**Figure 2:**
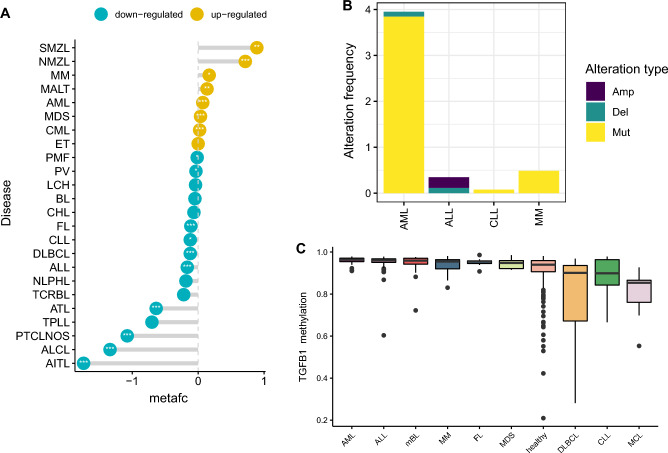
Landscape of expression and (epi)genetic alterations of *TGFB1* across blood cancer types. (**A**) Lollipop chart showing the difference of *TGFB1* expression between normal and tumor samples across cancer types in Pan-Hem-Diff. Blue depicts down-regulation in tumor and yellow depicts up-regulation in tumor. Asterisks represents the statistical *p* value (*p < 0.05; **p < 0.01; ***p < 0.001). (**B**) Genetic alteration frequencies of *TGFB1* across four blood cancers. **(C)** Box plot showing methylation levels of *TGFB1* across 9 blood cancer types and healthy controls (GSE28094).

To further investigate *TGFB1* expression across a broad subtypes of blood cancers, we retrieved 40 datasets with matched tumor and normal samples encompassing 22 blood cancer types (n = 9101, Pan-Hem-Diff cohort, Table S1). By performing a meta-analysis of datasets belonging to the same cancer type (Table S2, we observed that *TGFB1* were largely dysregulated in blood cancers. Myeloid malignancies like AML, MDS, and CML generally showed higher *TGFB1* expression when compared to normal samples and the opposite was seen in lymphoid leukemias (ALL and CLL) (Fig. 2A). We next investigated genetic alterations (including mutations, amplifications, and deletions) frequencies of *TGFB1* across blood cancers. The highest alteration frequency of *TGFB1* were observed in AML with “mutation” as the primary type (Fig. 2B). Copy number alteration (CNA) was the altered primary type in ALL with amplifications more commonly seen. In CLL and MM, mutations were the only genetic alteration (Fig. 2B). By analyzing the methylome data of *TGFB1* across 9 blood cancer types and healthy controls (GSE28094), we found only small variances of *TGFB1* methylation among common blood cancer types and normal samples (Fig. 2C). Furthermore, analyzing differential methylation patterns of *TGFB1* between tumor and normal samples revealed no differences in AML and DLBCL but hypermethylated *TGFB1* in MM (Figure S1B–E).

### Prognostic significance of *TGFB1* in hematological malignancies

**Figure 3:**
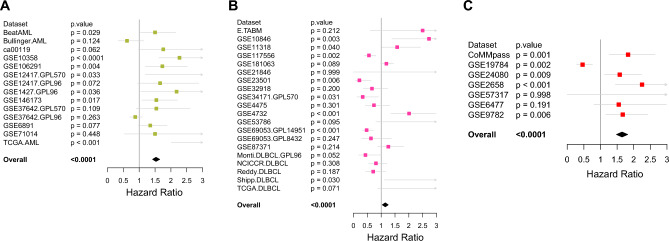
Prognostic Significance of *TGFB1* in hematological malignancies. (**A**–**C**) Forest plot showing hazard ratios (HRs) and *p*-values of *TGFB1* expression in each dataset among three blood cancers: AML (A), DLBCL (B), and MM (C). The hazard ratios and *p*-values were combined across datasets. The *p*-values were computed from Cox regression analysis in each dataset and combined using the weighted Z-method.

Our previous data reflects a hematologic-cancer-specific expression pattern of *TGFB1*. Following analyses will thus focus on *TGFB1* in hematologic cancers. We next sought to examine the prognostic significances of dysregulated *TGFB1* expression in three major blood cancer types (AML, DLBCL, and MM). We dichotomized high and low expression of *TGFB1* in each cohort by the Maxstat method. Univariable cox regression analysis was used to assess the prognostic impact of *TGFB1* in each dataset, followed by a meta-analysis of cox regression values (*p* values and HRs) inside each cancer type. Notably, *TGFB1* turned out to be an adverse prognosticator of OS for all three cancers (Fig. 3A–C). In summary, these results suggest that *TGFB1* was broadly dysregulated and significantly improved outcome predictions in blood cancers.

### Correlations between *TGFB1* expression and tumor microenvironment in hematological malignancies

**Figure 4:**
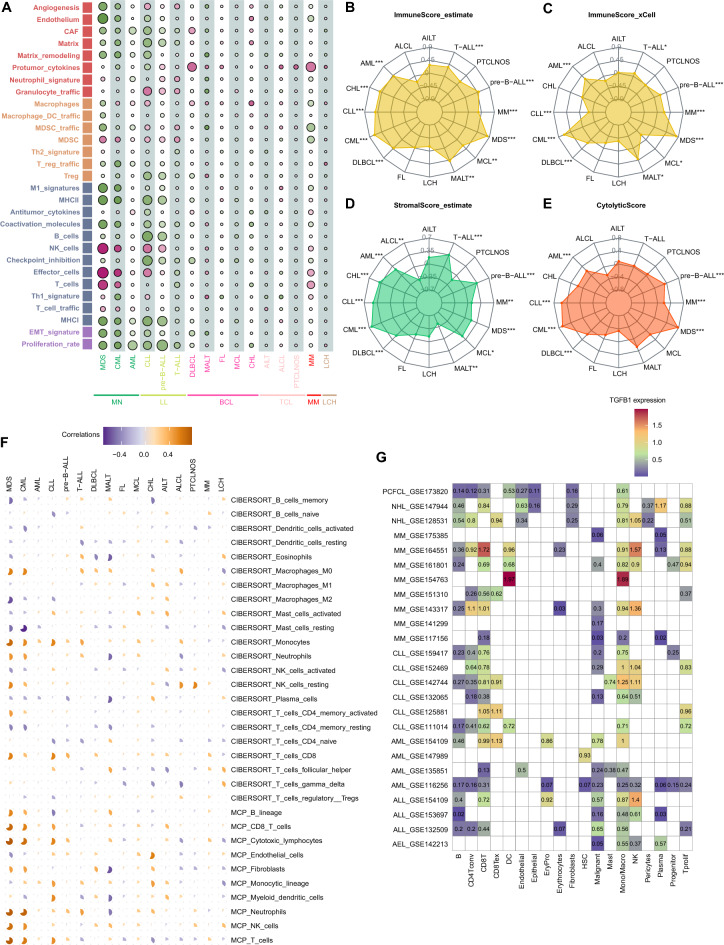
The relation between *TGFB1* expression with tumor microenvironment in hematological malignancies. (**A**) Bubble chart depicting the association between *TGFB1* expression and 29 TME signature scores across main blood cancer types in Hemap. (**B**–**E**) Radar chart showing correlations of *TGFB1* expression with immune, stromal, and cytolytic score across main cancer types in Hemap. Asterisks represents the statistical *p* value (*p < 0.05; **p < 0.01; ***p < 0.001). (**F**) Pie charts showing correlations of *TGFB1* expression with immune cell infiltration deconvoluted using CIBERSORT or MCP-counter algorithms in Hemap. Each pie chart indicates the correlation with each signature score in each cancer type. (**G**) Heatmap showing the expression levels of *TGFB1* in annotated cell types from scRNA-seq datasets.

As TGF-β signaling has been reported to be involved in the TME^18,19^, we continued to explore the correlations between *TGFB1* expression and 29 TME signature scores across hematological malignancies. In general, *TGFB1* expression was negatively correlated with stromal components such as angiogenesis, endothelium, CAFs, matrix, and matrix remodeling, especially in myeloid malignancies. In DLBCL and MM, a strong positive association was observed between *TGFB1* expression and pro-tumor cytokines; whereas in MDS and CML, *TGFB1* expression was positively correlated with tumor-suppressive components like NK and T cells and was negatively correlated with epithelial-to-mesenchymal transition (EMT) and tumor proliferation rate (Fig. 4A). This indicates context-specific roles of the TGF-β pathway across blood cancer types. We next examined the associations between *TGFB1* expression with immune and stromal scores as calculated by ESTIMATE. *TGFB1* expression was positively correlated with immune score in most blood cancer types, the most prominent ones being MDS and CML (Fig. 4B). This was true when immune score was calculated by xCELL algorism (Fig. 4C). *TGFB1* expression was positively correlated with the stromal score in AML, classical Hodgkin lymphoma (CHL), CLL, CML, DLBCL, mantle cell lymphoma (MCL), MDS, MM, pre-B ALL, and T-ALL (Fig. 4D). In addition, we also computed the cytolytic score for these blood cancers. The results again revealed strongest correlation between *TGFB1* expression and cytolytic score in CML and MDS (Fig. 4E). We then examined the correlation between *TGFB1* levels and the degree of immune cell infiltration in diverse blood cancers using both CIBERSORT and MCP-counter algorithms. As shown, *TGFB1* expression was positively associated with macrophages and monocytes infiltration in most cancers. Interestingly, *TGFB1* were positively correlated with the level of CD8 + T cells and NK cells but negatively correlated with that of CD4 + T cell (Fig. 4F). Furthermore, we investigated *TGFB1* expression at single-cell resolution in several blood cancer types. In line with previous results, *TGFB1* is highly expressed in monocytes/macrophages, NK and CD8 + T cells in these cancers (Fig. 4G).

We also performed Spearman correlation analysis to uncover the associations between *TGFB1* expression and immunomodulatory genes across blood cancers. We found that *TGFB1* was positively correlated with most of the immunomodulatory factors in myeloid and lymphoid leukemias as well as DLBCL but negatively correlated with most in MM (Figure S2). This indicates that *TGFB1* might play essential roles in the regulation of the immune response to these cancers.

### Gene set enrichment analysis of *TGFB1* reveals its association with the cancer immune response

**Figure 5:**
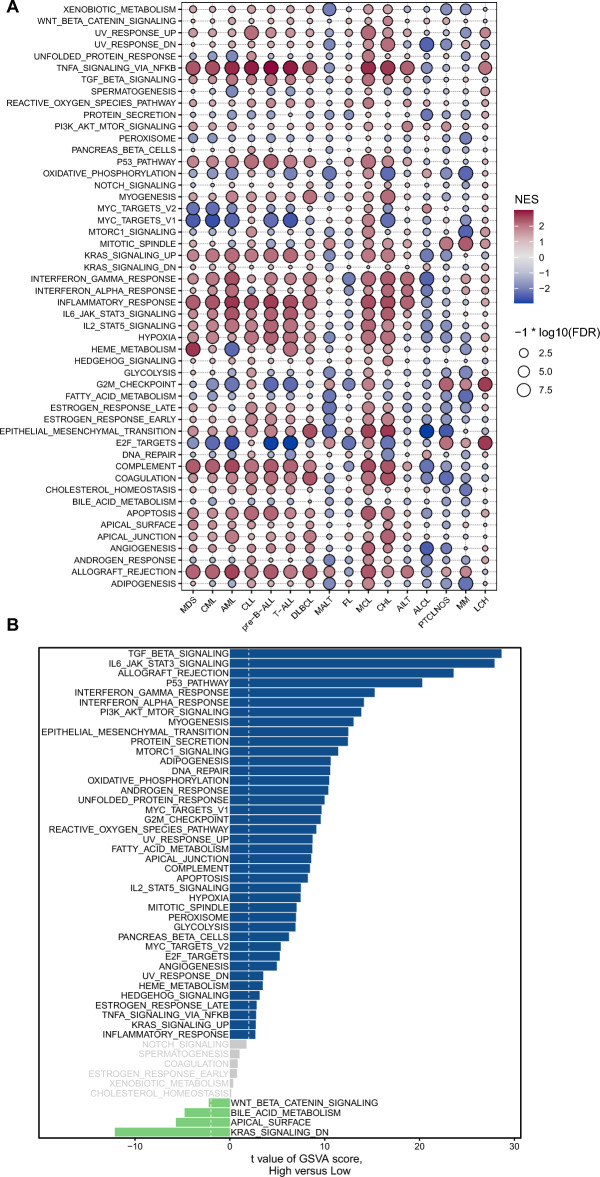
Relationships between *TGFB1* expression and Hallmark signaling pathways. (**A**) Enrichment analysis for Hallmark gene sets between blood cancer samples (Hemap) with high and low *TGFB1* expression (top 30% vs. bottom 30% of *TGFB1* expression). NES depicts the normalized enrichment score using the GSEA algorithm. (**B**) Differential activity of Hallmark gene sets between AML single cell samples (GSE116256) with high and low *TGFB1* expression (as stratified by the median expression value).

To explore the biological processes associated with *TGFB1* expression in blood cancers, we performed differential expression analysis between the top 30% *TGFB1* expression subgroup and bottom 30% *TGFB1* expression subgroup in each blood cancer type. Based on the differential expression genes (DEGs) between the high- and low- *TGFB1* subgroups, we performed GSEA analysis across blood cancer types to evaluate the *TGFB1*-associated cancer hallmarks. We found that immune-related pathways, such as TNFA-signaling-via-NFKB, TGF-BETA signaling, IFN-γ response, IFN-α response, inflammatory response, and allograft-rejection pathways were significantly enriched in most blood cancers. These results indicate that *TGFB1* might be actively participated in the TME and ligand–receptor interactions between malignant tumor cells and immune cells. Moreover, we also found that *TGFB1* was positively associated with the p53 pathway, KRAS signaling-up, and apical junction but was negatively associated with MYC targets, E2F targets, and G2M checkpoint in most cancers (Fig. 5A). Pathway analyses were also performed between single cells with high and low *TGFB1* expression, using scRNA-seq data from AML (GSE116256). As expected, TGF-BETA signaling was the top enriched pathway, followed by IL6-JAK-TAT3 signaling, allograft-rejection, p53 pathway, IFN-γ and IFN-α response. This agreed favorably with the results from bulk analyses. We also identified pathways negatively associated with *TGFB1*: KRAS signaling-down, apical surface, bile-acid metabolism, and WNT-BETA-CATENIN signaling (Fig. 5B).

### *TGFB1* predicts the response to cancer immunotherapy

**Figure 6:**
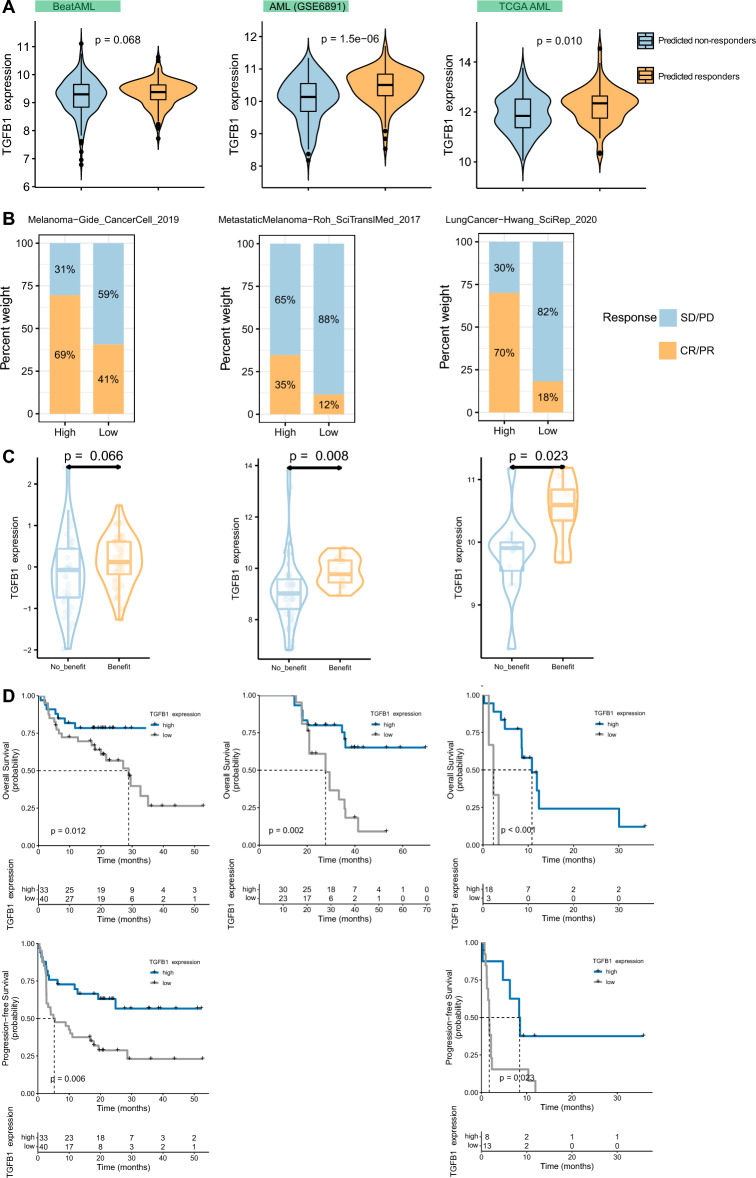
*TGFB1* expression predicts responses to immunotherapy (**A**) Violin plots comparing *TGFB1* expression between patients who benefit and who do not benefit from immunotherapy in AML, as predicted by the TIDE algorithm. (**B**) Bar plots showing percentages of responders (complete response [CR] or partial response [PR]) and non-responders (stable disease [SD] or progressive disease [PD]) to ICB among indicated ICB cohorts between patients with high and low *TGFB1* expression (as stratified by the median expression value). (**C**) Violin plots comparing *TGFB1* expression in responders (complete response [CR] or partial response [PR]) and non-responders (stable disease [SD] or progressive disease [PD]). (**D**) Kaplan–Meier curves depicting the OS and PFS of ICB-treated patients with high and low *TGFB1* expression. The optimal cut-off of *TGFB1* was determined by the Maxstat method. In (**B**–**D**), each vertical column corresponds to one ICB cohort as indicated in (**B**).

According to the clues mentioned above, we continued to explore the value of *TGFB1* as a tool to predict response to immunotherapy. We first calculated immune signature scores and predict immune checkpoint blockade (ICB) responses in datasets covering five blood cancer types using TIDE. Interestingly, we observed a consistent positive correlation between *TGFB1* expression and the dysfunction score but a negative correlation with M2 macrophages (Figure S3). Also, we found the predicted responders generally have significantly higher *TGFB1* expression than non-responders, especially in AML (Fig. 6A and Figure S4A). To test whether our findings could be generalized to solid tumors, we analyzed the predictive role of *TGFB1* in patients with cancer who received ICB therapy. The results showed that response rate of patients with high *TGFB1* expression was significantly higher than patients with low *TGFB1* expression and patients with a response to ICB had higher *TGFB1* expression than patients with no response, this could be applied to melanoma, lung cancer, and kidney cancer immunotherapy cohorts (Fig. 6B,C and Fig. S4B-C). Furthermore, we showed that patients with high *TGFB1* expression had an improved survival (both OS and PFS) than patients with low *TGFB1* expression (Fig. 6D and Figure S4D). Considering the type of immune checkpoint inhibitors, *TGFB1* remains predictive in cohorts treated with anti-PD1/PDL1 (Hwang_SciRep_2020, Ascierto_CancerImmunolRes_2016, Cho_ExpMolMed_2020, Du_NatCommun_2021, Lee_NatCommun_2020, and Liu_NatMed_2019), anti-CTLA4 (Roh_SciTranslMed_2017), or their combination (Gide_CancerCell_2019 and Miao_Science_2018) (Fig. 6B–D and Figure S4B-D). Overall, these data confirm the potential ability of *TGFB1* in predicting immunotherapy response across cancer types.

### Additional value of *TGFB1* expression in refining risk stratification in AML

**Figure 7:**
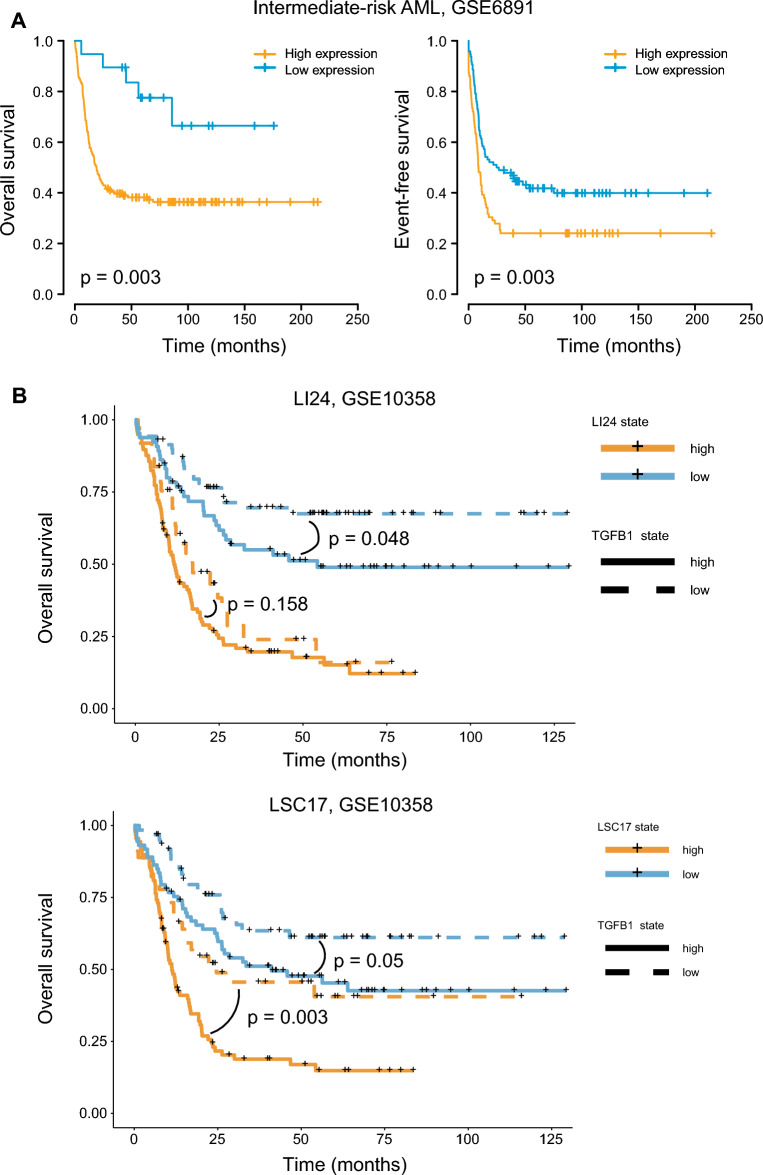
Additional value of *TGFB1* expression in refining risk stratification in AML. (**A**) OS and EFS according to *TGFB1* expression status among intermediate-risk patients from GSE6891. (**B**) OS of patients from GSE10358 as stratified by the LI24 and LSC17 score. Patients with a low- and high-risk score were further dichotomized by *TGFB1* expression status. The optimal cut-off of *TGFB1* was determined by the Maxstat method.

The prominent prognostic role of *TGFB1* expression prompted us to investigate if *TGFB1* may add prognostic value to the established prognostication systems. We first tested the prognostic value of *TGFB1* in intermediate-risk AML-a heterogeneous group of AML patients with various outcomes. In the GSE6891 cohort, the intermediate-risk group could be further dichotomized into two groups with remarkably different outcomes, for both OS and EFS (*P* = 0.003 for OS and *P* = 0.003 for EFS; Fig. 7A). We further tested the predictive value of *TGFB1* expression in the context of two gene expression-based prognostic models-LSC17 and LI24, which have shown their superior prognostic performance in risk stratification for AML patients^20,21^. In the GSE6891 cohort, *TGFB1* expression failed to further refine the two models (Figure S5). However, in the GSE10358 cohort, *TGFB1* status could dichotomize survival in the LI24 low-risk group and it could still discriminate between shorter and longer OS both within the LSC17 high- and low-risk groups (Fig. 7B and C). These results suggest *TGFB1* as a potential candidate for refining existing classification schemes.

### Clinical correlation analysis of *TGFB1* in AML

**Figure 8:**
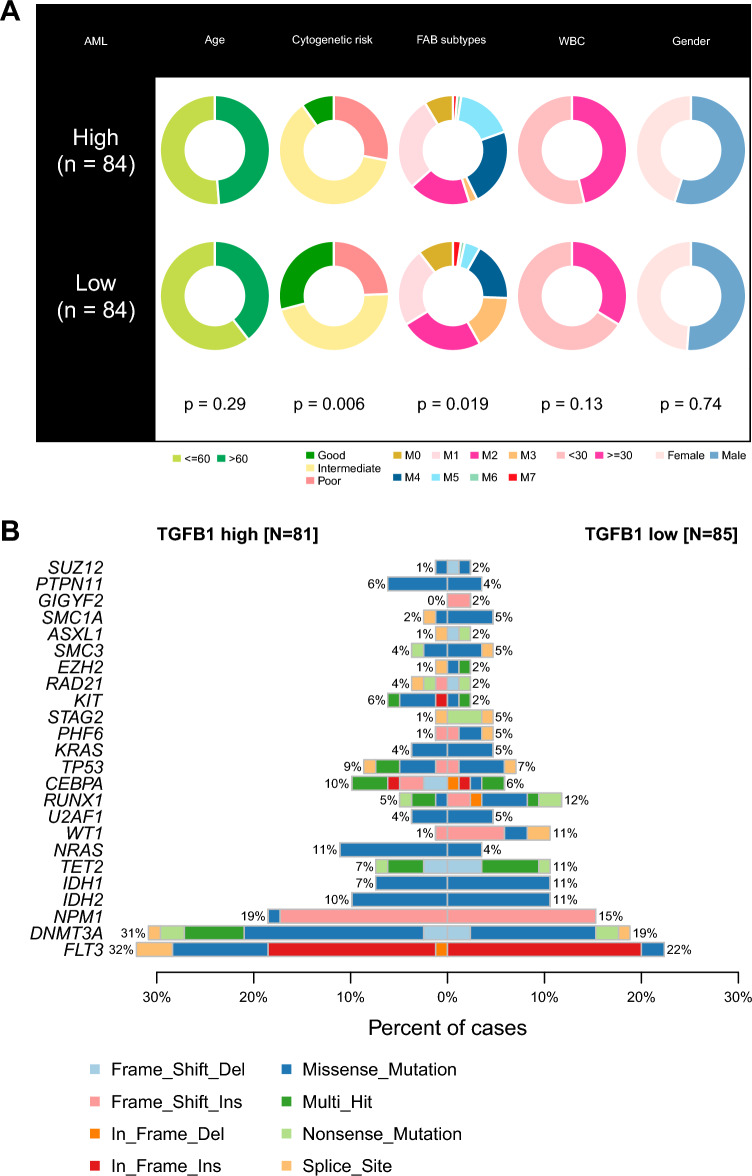
*TGFB1* expression correlates with distinct clinical parameters and mutation profiles in AML. (**A**) Pie charts showing the Chi-squared test results of clinical parameters for *TGFB1* status using the median expression as the cut off. (**B**) Co-bar plots showing the comparison of mutational profiles between patients with high and low *TGFB1* expression (as stratified by the median expression value) in the TCGA AML dataset.

Our results suggests that *TGFB1* might perform a key role in the pathogenesis of AML. We continued to examine the associations between *TGFB1* expression and the clinical and genetic characteristics in the TCGA AML cohort. We found an association between *TGFB1* expression and the risk classification in AML: patients with high *TGFB1* expression were more frequently classified in the intermediate risk group and less frequently in the favorable risk group than patients with low *TGFB1* expression. For French-American-British (FAB) classification of AML, more M4 and M5 subtypes and less M2 and M3 subtypes were presented in patients with high *TGFB1* expression (Fig. 8A). No significant associations were found between *TGFB1* expression and other clinical parameters, although patients with high *TGFB1* expression tended to have higher white blood cell (WBC) counts than those with low *TGFB1* expression (Fig. 8A). To determine whether *TGFB1* correlated with distinct mutational profiles characterized for AML, we identified significantly mutated genes that occurred in patients with high and low *TGFB1* expression. As shown in Fig. 8B, patients with high *TGFB1* expression more frequently harbored *NRAS* and *DNMT3A* mutations and less frequently harbored *WT1* mutation (Fig. 8B).

## Discussion

*TGFB1* encodes a secreted ligand of the TGF-β1 superfamily of proteins. TGF-β1 could bind various TGF-beta receptors leading to recruitment and activation of SMAD family transcription factors that regulate gene expression. This protein has been shown to execute seemingly contradictory roles in both normal and malignant states^3^. In this study, we first analyzed *TGFB1* expression across normal and malignant tissue/cell types. Interestingly, we observed a hematologic-tissue-specific expression pattern of *TGFB1*. Indeed, *TGFB1* has been shown to be an important regulator of hematopoietic lineage determination and *TGFB1* dysregulation has been found in various hematologic malignancies^12^. Accordingly, when comparing *TGFB1* expression between tumor and normal samples encompassing 22 blood cancer types, we found *TGFB1* to be largely dysregulated in blood cancers: it was generally up-regulated in myeloid malignancies like AML, MDS, and CML and down-regulated in lymphoid leukemias (ALL and CLL) when compared to normal samples. Interestingly, the expression of *TGFB1* appears to be increased in low-risk lymphomas, whereas its expression appears to be decreased in Peripheral T-cell lymphoma (PTCL) (Fig. 2A). It should be mentioned that these analyses were performed in different datasets that reflect bulk expression states of respective tumors. Therefore, the differential expression of *TGFB1* might be contributed not only by malignant cells, but also immune/stromal cells from the TME. Also, previous findings reported that the expression of *TGFB* signaling components could be either up-regulated or down-regulated in lymphomas, depending on the context, histological subtype, and stage of development^22^. It is highly recommended that future studies could examine *TGFB1* expression in specific subtypes of lymphomas and at a single-cell level. Previous studies have reported somewhat contradictory roles of the TGF-β1 pathway across blood cancer types. For example, Xu et al. found that TGF-β1 exerted pro-survival effects in myelo-monocytic leukemia cells^23^. Whereas another study by Wu et al. reported that TGF-β1 mRNA expression levels were significantly down-regulated in leukemic cells compared with normal CD34 + cells. Transfection of the TGF-β1 gene to leukemia cells induced cell apoptosis and inhibited cell proliferation^24^. These opposing results of TGF-β were probably due to the context-dependent effects of this gene. In our analyses, we showed that high *TGFB1* expression was more commonly seen in the M4 (acute myleomonocytic leukemia) and M5 (acute monocytic leukemia) FAB subtypes (Fig. 8A), which agreed favorably with the preferential expression of *TGFB1* in monocytes (Figure S1A). This observation together with Xu et al. indicated a potential subtype-specific role of TGF-β1 in leukemogenesis. We also observed that patients with high *TGFB1* expression more frequently harbored *DNMT3A* mutations. Interestingly, previous study has reported that high TGF-β1 expression correlated with increased expression of *DNMTs*^25^. The functional link between two gene alterations awaits further investigation.

To further dissect the overall effects of *TGFB1* in blood cancers, we investigated the prognostic values of dysregulated *TGFB1* expression in three major blood cancer types (AML, DLBCL, and MM). Remarkably, higher *TGFB1* expression negatively correlated with patient outcomes in all three types of cancers by performing meta-analyses. In line with this, high TGFβ levels were shown to be generally associated with poor prognosis in solid tumors^26–28^. The reason that *TGFB1* adversely impacts prognosis might be: first, TGFβ1 binds its receptors, activating the downstream signaling pathway and regulating the tumor-promoting gene-expression programs; second, TGFβ1 signaling could lead to a pro-tumoral niche by reprogramming stromal components and suppressing immune cells, as evidenced by the finding that *TGFB1* expression was strongly positively correlated with pro-tumor cytokines in DLBCL and MM. We also noticed that *TGFB1* expression was positively associated with CAF signature in DLBCL (Fig. 4A). Thus, it is reasonable to hypothesize that targeting TGF-β signaling could disrupt these pro-tumor components and benefit patients. It should be noted that the prognostic impact of *TGFB1* expression was evaluated using bulk expression profile data, which averages the diverse cells within each tumor, making the percentage of tumor cells hard to determine and masking the influences of TME. We therefore analyzed the correlations between *TGFB1* expression and TME in hematological malignancies. In MDS and CML, *TGFB1* expression was positively correlated with tumor-suppressive components like NK and T cells but was negatively correlated with EMT and tumor proliferation rate. In CML, it was reported that TGF-β signaling was repressed by the Evi-1 oncoprotein to facilitate this disease into blast crisis^29,30^. On the other hand, it was shown that inhibiting TGFβ1 signaling could improve anemia in MDS patients^31,32^. That said, these studies provided little information about how TGFβ1 might impact the TME of these diseases. Further analyses revealed that *TGFB1* expression was positively correlated with immune score and cytolytic score in MDS and CML. Our results indicated that TGFβ1 might induce a protective niche with enriched immune cells in MDS and CML patients. However, future functional studies were warranted to test this hypothesis.

TGF-β1 and its binding proteins have been proposed as promising targets of therapies for different blood cancers. For example, modulating TGFβ1 signaling could be utilized to improve the ineffective erythropoiesis in MDS^31,32^. Also, since TGF-β1 is a strong immunosuppressor, existing data suggests that TGFβ1-inhibitory therapies could restore cancer immunity and even synergize with other immunotherapies^3^. In a study by Tauriello et al., researchers developed human-like mouse models of metastatic colorectal cancer (CRC) and show that TGFβ1 inhibition could synergize with anti-PD-L1 therapy to exert robust T cell responses against metastatic disease^8^. Another study has also shown that TGFβ1 inhibition facilitated T-cell penetration and improved the outcomes of anti-PD-L1 treatment using experimental models^9^. These findings prompted us to explore whether *TGFB1* expression could be used as a tool to predict response to immunotherapy. We first predicted ICB responses in datasets covering five blood cancer types using TIDE and found that predicted responders generally have significantly higher *TGFB1* expression than non-responders, especially in AML. Importantly, further evaluating using real-world ICB datasets revealed that patients with high *TGFB1* expression generally had higher response rate and improved survival (both OS and PFS) for immunotherapy than those with low *TGFB1* expression. However, these results were somewhat conflicting with previous reports that enriched TGFβ1 signaling in stromal cells were associated with primary resistance to immunotherapy^33,34^. It should be mentioned that both studies used signatures derived from TGFβ1-activated CAFs to evaluate patients’ response to immunotherapy. Whereas our results were based on bulk tumor transcriptomic profiling cohorts and the predictive value was focused on a single gene, which can hardly reflect the complex networks of TGFβ1 signaling and cell-to-cell communication. Future prospective immunotherapy studies regarding both the mRNA and protein aspects of TGFβ1 are warranted. Further, it is useful to construct TGFβ1-specific gene signatures to predict immunotherapy responses.

In summary, we performed the first comprehensive analysis of *TGFB1* across blood cancers. We found that *TGFB1* expression were broadly dysregulated in blood cancers and generally associated with adverse prognosis. Additionally, *TGFB1* expression were associated with distinct TME properties among different blood cancer types. Our results also suggest that *TGFB1* expression could be a useful marker to predict immunotherapy responses. Functional and mechanistic studies are needed to further understand the role of TGFβ1 signaling in blood cancers.

### Supplementary Information

Supplementary Figures.

Supplementary Table S1.

Supplementary Table S2.

## Data Availability

The datasets analyzed in this study are available in the following open access repositories: GTEx, www.gtexportal.org/, CCLE, https://www.broadinstitute.org/ccle , TCGA, https://portal.gdc.cancer.gov/, UCSC Xena, https://xena.ucsc.edu , cBioPortal, http://www.cbioportal.org, GEO, https://www.ncbi.nlm.nih.gov/geo/ (GEO accession numbers: please refer to Table S1 for details), GDC data portal, https://portal.gdc.cancer.gov/, PRECOG, https://precog.stanford.edu/ , Hemap data, https://www.synapse.org (https://doi.org/10.7303/syn21991014), TISCH2, http://tisch.comp-genomics.org/home/, TIDE, http://tide.dfci.harvard.edu/, MSigDB, http://www.broad.mit.edu/gsea/msigdb. Other data used to support the findings of this study are available from the corresponding author upon request. All authors agree to publish.
