# Supplementary material for: Comprehensive characterization of TGFB1 across hematological malignancies
Source: Sci Rep. 2023 Nov 4;13:19107. doi: 10.1038/s41598-023-46552-8 (PMC10625629; doi:10.1038/s41598-023-46552-8)
Supplement: Supplementary file 1 — Supplementary Figures. [file 41598_2023_46552_MOESM1_ESM.docx]

**Supplementary Figures**


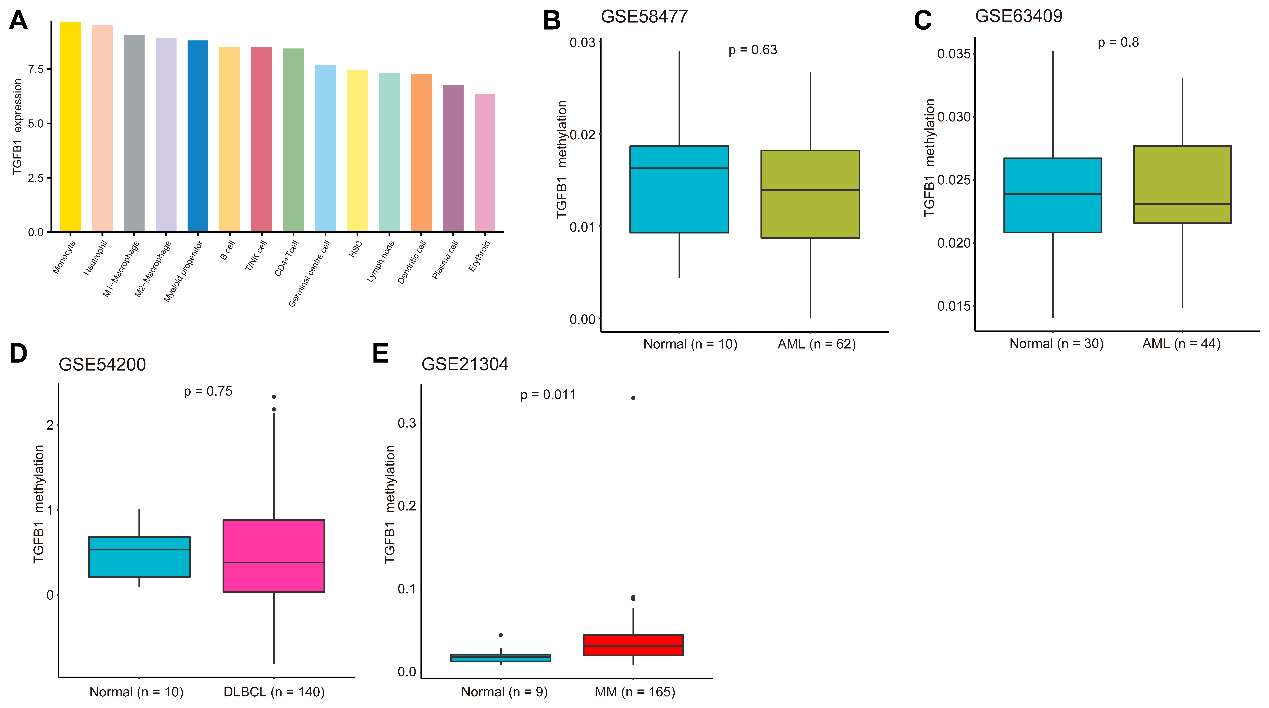


**Figure S1. Landscape of expression and epigenetic alterations of TGFB1 across blood cancer types. (A)** Bar plot showing *TGFB1* expression in normal cell populations from the Hemap dataset. **(B-E)** Boxplots comparing methylation levels of *TGFB1* between tumor and normal samples in three blood cancer types.


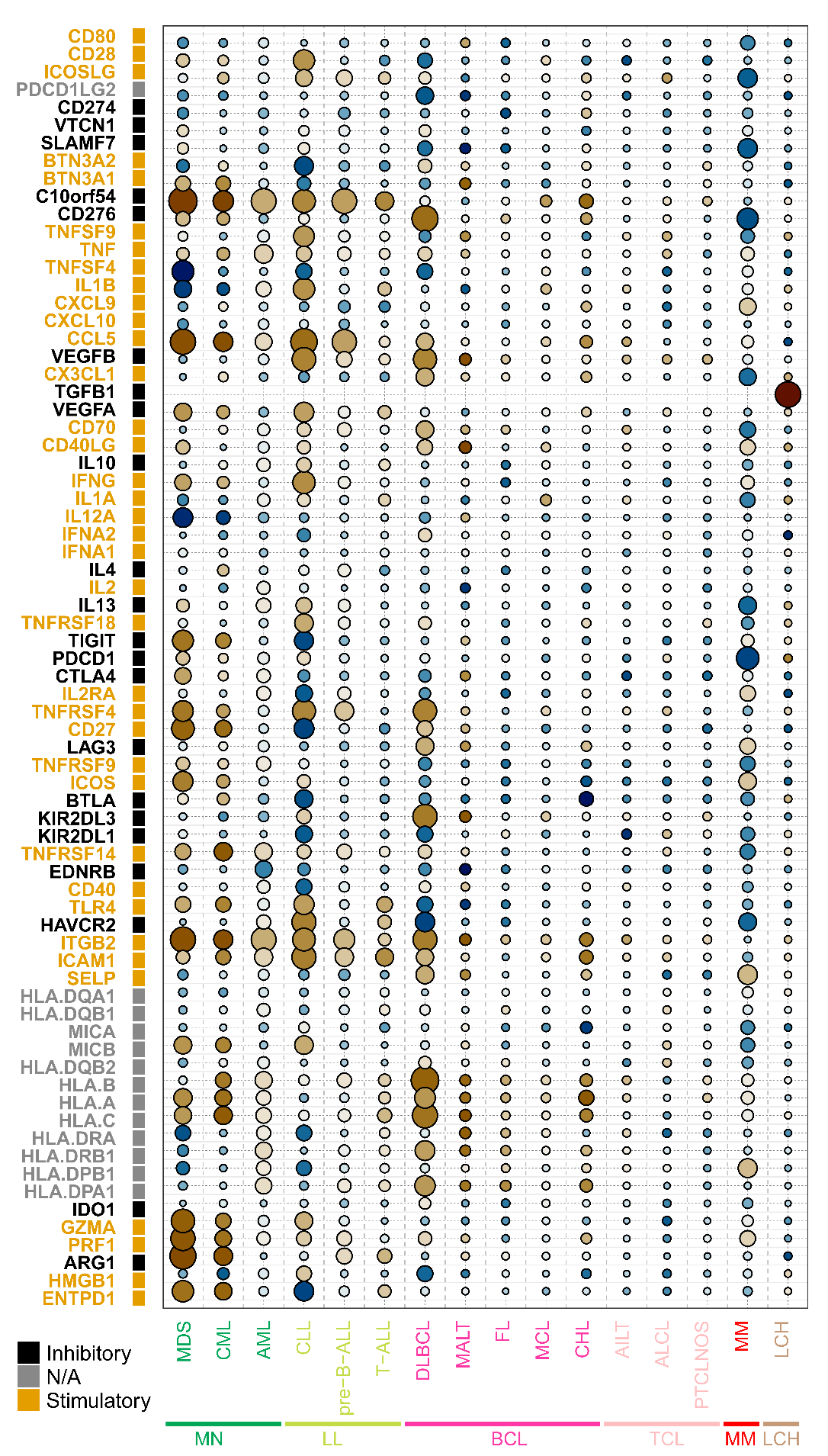


**Figure S2. Bubble chart depicting the association between TGFB1 expression and immunomodulatory genes across main cancer types in Hemap.**


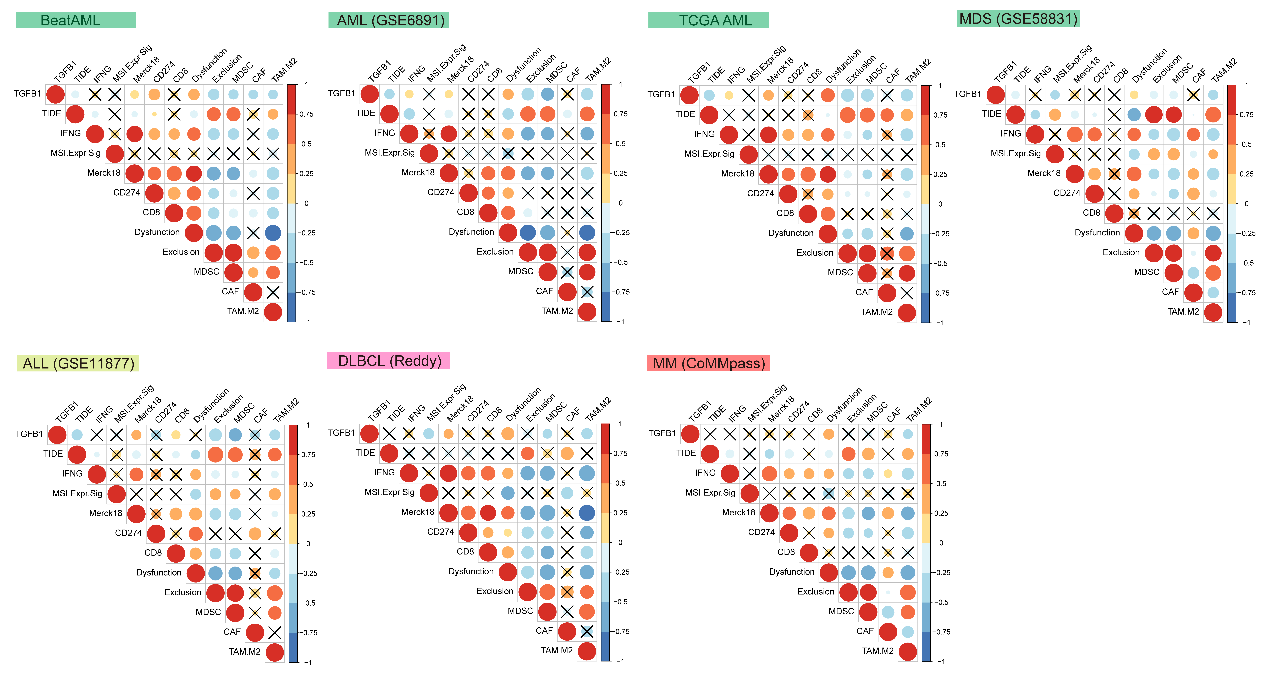


**Figure S3.** **Correlograms showing the association between TGFB1 expression with immune signature scores in indicated blood cancer types, as calculated using the TIDE method.**

**
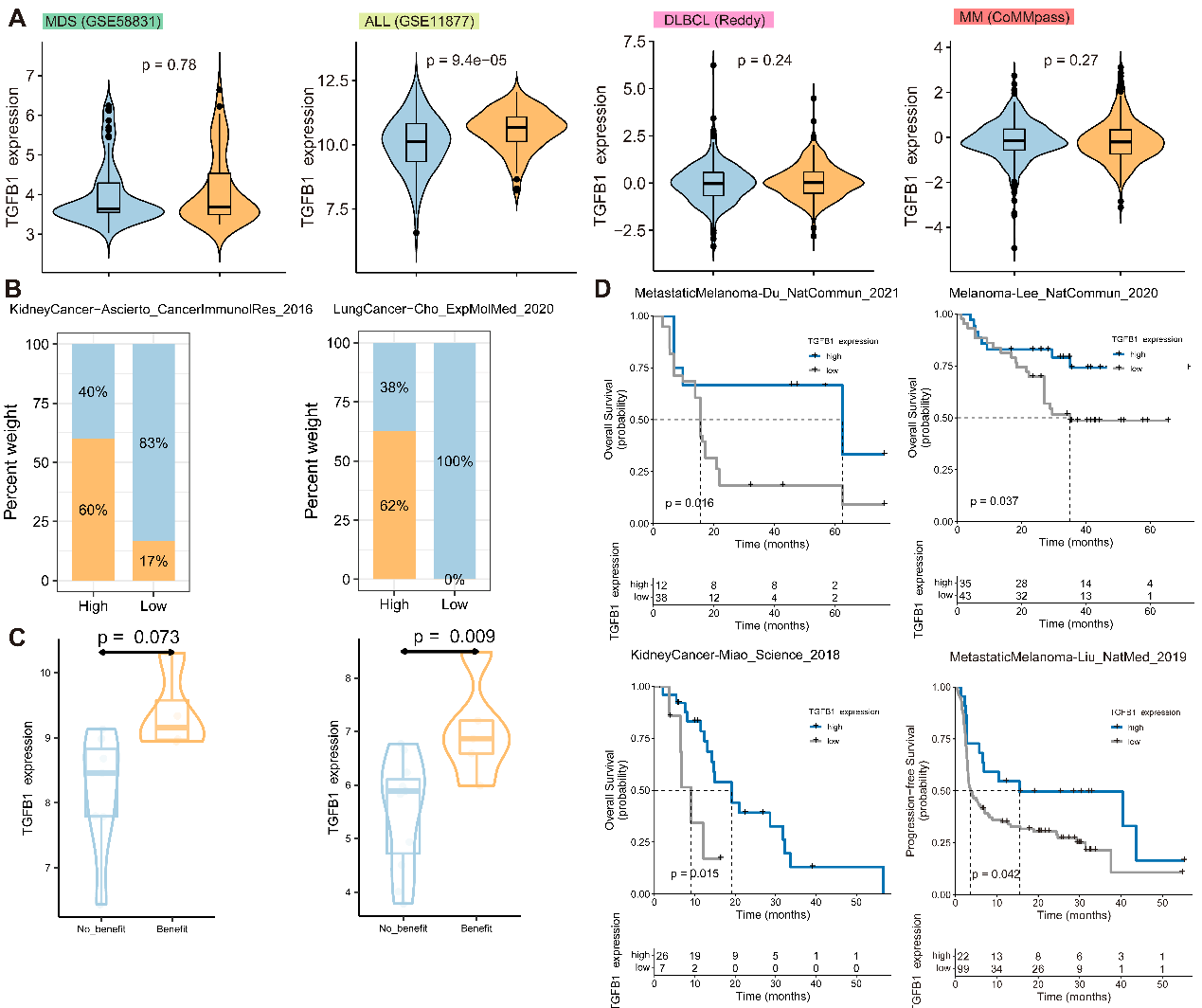
**

**Figure S4.** ***TGFB1* expression predicts responses to immunotherapy (A)** Violin plots comparing *TGFB1* expression between patients who benefit and who do not benefit from immunotherapy in four blood cancer types, as predicted by the TIDE algorithm. **(B)** Bar plots showing percentages of responders (complete response [CR] or partial response [PR]) and non-responders (stable disease [SD] or progressive disease [PD]) to ICB among indicated ICB cohorts between patients with high and low *TGFB1* expression. **(C)** Violin plots comparing *TGFB1* expression in responders (complete response [CR] or partial response [PR]) and non-responders (stable disease [SD] or progressive disease [PD]). **(D)** Kaplan-Meier curves depicting the OS and PFS of ICB-treated patients with high and low *TGFB1* expression. In **(B-C)**, each vertical column corresponds to one ICB cohort as indicated in **(B)**.

**
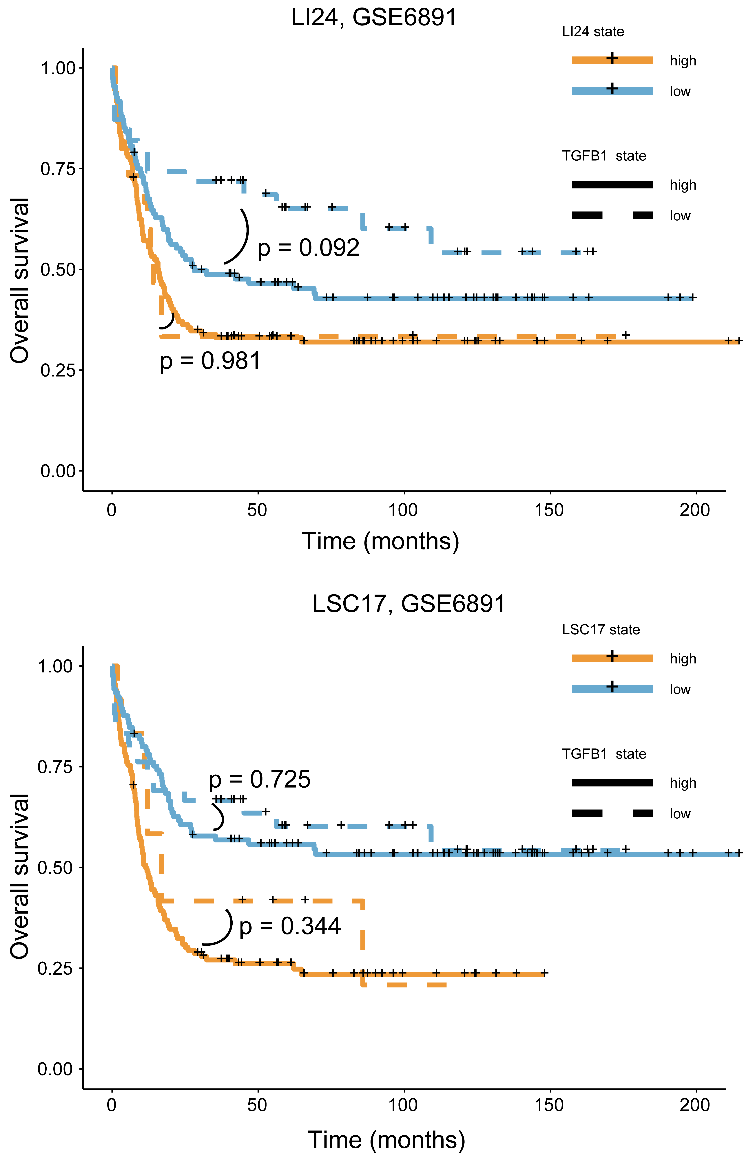
**

**Figure S5.** **Additional value of *TGFB1* expression in refining risk stratification in AML.** OS of patients from GSE6891 as stratified by the LI24 and LSC17 score. Patients with a low- and high-risk score were further dichotomized by *TGFB1*expression status.
